# Postoperative anterior cruciate ligament rehabilitation: A survey in Gauteng, South Africa

**DOI:** 10.4102/sajp.v81i1.2144

**Published:** 2025-08-20

**Authors:** Colett Robbertse, Vaneshveri Naidoo

**Affiliations:** 1 Department of Physiotherapy, Faculty of Health Sciences, University of the Witwatersrand, Johannesburg, South Africa

**Keywords:** post-operative ACL rehabilitation, ACL rehabilitation, ACL rehabilitation protocols, ACL rehabilitation management strategies, ACL rehabilitation modalities

## Abstract

**Background:**

Post-operative rehabilitation is key to successful outcomes in anterior cruciate ligament (ACL) surgery. This study aimed to determine the management strategies (accelerated vs. non-accelerated protocols) and treatment modalities used by physiotherapists for post-operative ACL rehabilitation in Gauteng, South Africa.

**Objectives:**

This study determined the frequency of accelerated and non-accelerated protocols, identified rehabilitation objectives, commonly used physiotherapy modalities and exercise recommendations within the first 6 weeks of post-operative ACL rehabilitation.

**Method:**

A cross-sectional study was carried out between 15 December 2021 and 27 May 2022, using a custom-designed questionnaire. The survey was initially distributed to members of the South African Society of Physiotherapy (SASP) and Physiotherapy Association of South Africa (PASA), as well as shared on social media platforms. Data collection was completed in 6 months. Descriptive statistics (frequencies means and percentages) were analysed.

**Results:**

Out of 120 responses received, 47% (56) were analysed. Management strategies included the accelerated and non-accelerated programmes: 70% (39) of the participants used the accelerated protocol and 23% (13) used the non-accelerated protocol. Furthermore, 59% used protective bracing as part of their management and 66% (37) started rehabilitation post-operatively. Treatment modalities used included myofascial release (75%, 41), peripheral joint mobilisation (63%, 35), massage (57%, 32) and cryotherapy (34%, 19).

**Conclusion:**

Remarkable variation was found in treatment protocols, modalities and exercise prescription.

**Clinical implications:**

Using clinical evaluation, objective outcome measures and functional tests as an objective criterion is crucial in decision-making regarding return to function and sport.

## Introduction

Anterior cruciate ligament (ACL) injuries are prevalent across various age groups and activity levels, significantly affecting knee joint stability and functional movement (Shea & Carey 2015). The ACL plays crucial role in stabilising the knee joint, preventing excessive anterior tibial movement and controlling rotational forces during weight-bearing activities (Petersen & Tillmann 2002; Robbertse 2023; Sakane et al. 1999). Injuries to the ACL commonly result from non-contact mechanisms such as sudden directional changes or hyperextension, often occurring in sporting environments (Boden et al. 2010; Mclean 2007; Robbertse 2023; Singh 2018). Surgical intervention is generally the preferred treatment for complete ACL ruptures, as it aims to restore knee functionality and prevent long-term joint instability (Shea & Carey 2015).

Post-operative rehabilitation plays a critical role in ensuring successful recovery and return to pre-injury activity levels. However, significant variability exists in rehabilitation protocols, leading to inconsistencies in recovery outcomes (Janssen et al. 2018). While some approaches advocate for early mobilisation, others follow more conservative strategies that delay weight-bearing progression (Markatos et al. 2013). This lack of consensus contributes to differing timelines for functional recovery and return to sports (Eitzen et al. 2010; Robbertse 2023; Yabroudi & Irrgang 2013). Notably, Greenberg et al. (2018) reported that only 33% of athletes resume sports within 1 year post-injury, while 37% never regain their pre-injury participation levels. These findings highlight the importance of optimising rehabilitation strategies to improve recovery outcomes and reduce the risk of re-injury.

The debate surrounding the most effective rehabilitation approach has led to two primary protocols: accelerated and non-accelerated rehabilitation. The traditional non-accelerated protocol, established by Paulos et al. (De Carlo et al. 1992), involves immobilising the knee joint at a flexion angle of 30º for a period of 2 weeks, followed by the application of a hinged brace. Patients were prohibited from WB for 8 to 10 weeks. They steadily progressed to partial weight-bearing (PWB); full weight bearing (FWB) ensued after 12–14 weeks with isokinetic exercises simultaneously initiated. If patients achieved full range of motion (ROM) and satisfactory strength within 9 to 12 months, they were permitted to resume normal activities. Interestingly though, during the era of this protocol, patients who did not comply with the WB and exercise guideline by moving sooner achieved better outcomes with fewer complications. These findings and complications, such as reduced knee ROM and increased scar tissue formation, prompted the development of accelerated protocols. Andrade et al. (2020) and Shelbourne, Klootwyk and De Carlo (1992) demonstrated that accelerated rehabilitation, including early weight-bearing and progressive ROM exercises, yields superior results in strength, stability and functional recovery.

Despite advancements in rehabilitation strategies, a lack of standardisation persists. A wide range of protocols, strategies and modalities are used by physiotherapists in ACL rehabilitation following surgery, leading to variability in practice (Cristiani et al. 2021; Eitzen et al. 2010; Makhni et al. 2016; Robbertse 2023; Yabroudi & Irrgang 2013). This variability arises from individual practitioners developing their own protocols in collaboration with orthopaedic surgeons and drawing from their clinical expertise. However, this disparity between empirical evidence and the findings in the literature can result in inconsistencies that have also been noted globally (Aquino et al. 2021; Dingenen et al. 2021; Ebert et al. 2019; Fausett Reid & Larmer 2022; Greenberg et al. 2018; Korakakis et al. 2021; Robbertse 2023). Consequently, this variability may delay recovery of functional independence or return to sport and, in some cases, hinder patients from resuming sports activities altogether (Korakakis et al. 2021; Robbertse 2023). Furthermore, factors such as pre-operative rehabilitation, protective bracing, early mobilisation and specific exercise prescriptions all influence recovery trajectories (Andrade et al. 2020; Robbertse 2023; Wilk & Arrigo 2016).

This study investigates the management strategies and treatment modalities physiotherapists use for post-operative ACL rehabilitation in Gauteng, South Africa (Robbertse 2023). Specifically, it explores the following objectives: to determine the use of accelerated and non-accelerated protocols; to identify specific rehabilitation objectives (when rehabilitation starts, the WB protocol, the use of protective bracing, the time frame or period when patient return to normal function and sport); to identify the frequently used physiotherapy modalities and exercise prescription during the initial 6 weeks of post-operative ACL rehabilitation (Robbertse 2023). By examining these factors, the research aims to contribute to a more standardised and evidence-based approach to ACL rehabilitation, ultimately improving recovery outcomes and minimising long-term functional limitations.

## Research methods and design

This was a cross-sectional, quantitative study design using a self-administered questionnaire (Online Appendix 1) to collect data. The participants were qualified physiotherapists in Gauteng, South Africa, who were involved in post-operative ACL rehabilitation. Convenience sampling was used to select the participants, who were sourced from registered physiotherapists affiliated with the South African Society of Physiotherapy (SASP) and the Physiotherapy Association of South Africa (PASA) (Robbertse 2023).

For this study, a questionnaire was developed from the literature (Andrade et al. 2020; Filbay & Grindem 2019; Robbertse 2023; Wright et al. 2008a, 2008b). Their views shed light on various treatment strategies employed in post-operative ACL rehabilitation. The questionnaire consisted of three sections: demographic information (Part 1), questions about ACL rehabilitation protocols (Part 2) and physiotherapy strategies, modalities and exercise recommendations (Part 3).

To improve content validity, a pilot study was conducted, involving five experts with expertise in academic and clinical aspects of post-operative ACL rehabilitation. Their feedback and suggestions were considered, and the final version was checked by the experts before proceeding with data collection.

REDcap, a secure web application for online surveys, was used to distribute the questionnaire. Participants who agreed to participate in the study were directed to the survey page, which included introduction, explanation, ethical approval details and survey instructions. The link to the survey was distributed by the SASP and PASA, among their members (Robbertse 2023).

Given the low response rate, an ethics amendment approval was obtained to post the link to the study on social media platforms such as WhatsApp, Facebook and Twitter to encourage participation from physiotherapists. The study advertisement was subsequently shared using social media (WhatsApp) with physiotherapy groups in Gauteng. Additionally, the SASP shared the link of the study on their social media platforms (Robbertse 2023). The data collection phase concluded after 6 months.

The data were captured on Excel, and descriptive statistics such as frequencies, percentages and means were calculated.

### Ethical considerations

Ethical clearance was granted by the University of the Witwatersrand Human Research Ethics Committee, Faculty of Health Sciences on 23 August 2021 (protocol number M210620) (Robbertse 2023). Participants were anonymous; there were no identifying data collected on the survey. Informed consent for participation was obtained, and the participants could withdraw from the survey at any time. The PASA and SASP databases are strictly confidential. POPIA legislation was adhered to.

## Results

Out of the 120 physiotherapists who accessed the questionnaire on REDcap, 22 responses were incomplete, and 42 responses were excluded as the participants were located outside of the Gauteng province (Robbertse 2023). This resulted in a total of 56 (47%) completed questionnaires from physiotherapists in Gauteng that were analysed.

Table 1 provides an overview of the physiotherapy protocol used – accelerated vs non-accelerated, including the alignment with the respective definitions.

**TABLE 1:** Accelerated versus non-accelerated anterior cruciate ligament rehabilitation protocols (*N* = 56).

Rehabilitation protocol utilised	*N*	%	Alignment to definition	
			*n*	%
Accelerated	39	70	9	23
Non-accelerated	13	23	1	8
Other	4	7	-	-

**Total**	**56**	**100**	-	-

*Source*: Adapted from Robbertse, C., 2023, ‘Physiotherapy management of post-operative ACL Rehabilitation: A cross-sectional survey in Gauteng, South Africa’, Master’s thesis, University of the Witwatersrand, p. 56, viewed from https://hdl.handle.net/10539/37801

The accelerated protocol for ACL rehabilitation was used by the majority of the participants. The category ‘other’ chosen by participants described the following: ‘Depending on the surgeon and graft used, I utilise rehabilitation using either one or the other protocol’.

The protocol serves as a guideline, and patient adjustments are made based on their progress during rehabilitation. If the patient meets the progression criteria earlier than expected, they are accelerated; otherwise, they are not. Factors such as the surgical procedure and the surgeon’s advice also contribute to the decision-making process regarding the protocol type. (Robbertse 2023:56)

‘Accelerated is my preference, but surgeons may specify non-accelerated’, and ‘It depends on the surgeon, as different protocols may be utilised’.

**FIGURE 1:**
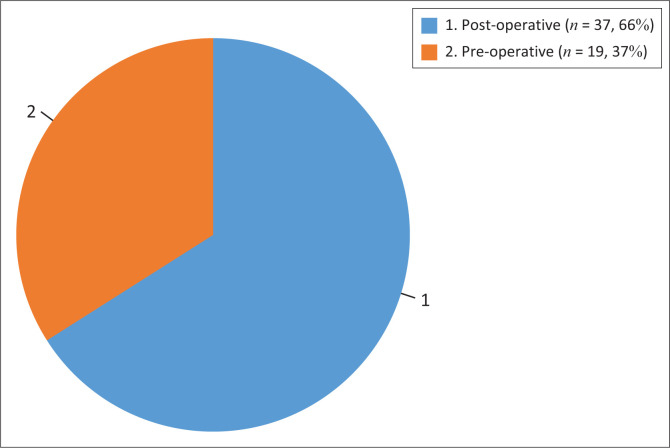
Number of physiotherapists starting rehabilitation preoperatively and post-operatively (*N* = 56).

Figure 1 illustrates that rehabilitation commenced post-operatively by most.

Table 2 illustrates the WB regimen reported by the participants. Most of the participants indicated full weight-bearing as soon as pain allows. The category ‘other’ included the following responses: ‘Partial PWB for 2 weeks, followed by FWB with a brace for 6 weeks’, ‘WB depends on the surgical intervention and the surgeon’s recommendation, as well as the individual’s pain tolerance’, ‘PWB for 2–4 weeks, then progressing to WB as tolerated based on pain levels’, and ‘PWB for 0–2 weeks, followed by FWB from 2 to 6 weeks’.

**TABLE 2:** Weight-bearing protocol, 0–6 weeks (*N* = 56).

Weight-bearing protocol (0–6 weeks)	*n*	%
Full weight-bear (FWB) as soon as pain allows	31	55
Partial weight-bearing (PWB)	20	36
Non-weight bear (NWB)	1	2
Other	4	7

**Total**	**56**	**100**

*Source*: Adapted from Robbertse, C., 2023, ‘Physiotherapy management of post-operative ACL Rehabilitation: A cross-sectional survey in Gauteng, South Africa’, Master’s thesis, University of the Witwatersrand, p. 57, viewed from https://hdl.handle.net/10539/37801

**FIGURE 2:**
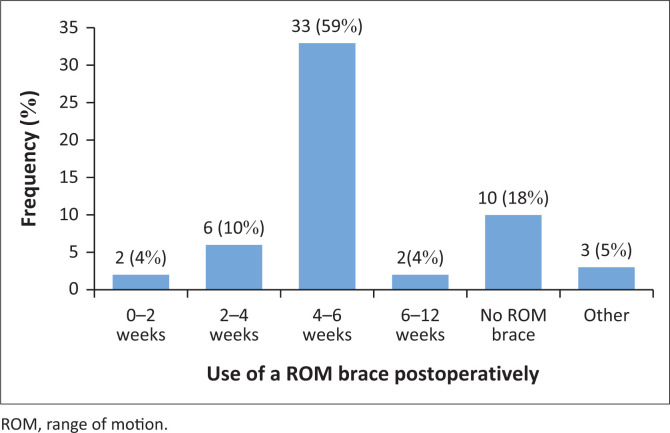
Use of a range of motion brace protocol post-operatively (*N* = 56).

Figure 2 illustrates the post-operative ROM brace protocol. Most participants used the ROM brace for 4–6 weeks.

Under ‘other’, the following was noted: ‘Brace usage is mostly avoided unless the surgeon deems it necessary for additional stability in the initial weeks’, ‘Brace usage is dependent on the surgeon. One surgeon does not prescribe a brace at all, while others may use it for up to 6 weeks’, and:

Brace usage is sometimes dependent on the surgeon. If it is my preference, it is determined on a case-by-case basis, considering factors such as biomechanics and movement control rather than adhering to a specific time frame. (Robbertse 2023:58)

Table 3 illustrates that most patients resumed function from 6 to 12 weeks and returned to sport 6–9 months post-operatively.

**TABLE 3:** Return to function and sport (*N* = 56).

Return period to normal function and sport	Return to function		Return to sport	
	*n*	%	*n*	%
0–6 weeks	6	11	-	-
12–18 weeks	19	34	-	-
6–12 weeks	22	39	-	-
0–6 months	-	-	4	7
6–9 months	-	-	22	39
9–12 months	-	-	16	29
Other	-	-	3	5
Determined by functional test	9	16	11	20

**Total**	**56**	**100**	**56**	**100**

*Source:* Adapted from Robbertse, C., 2023, ‘Physiotherapy management of post-operative ACL rehabilitation: A cross-sectional survey in Gauteng, South Africa’, Master’s thesis, University of the Witwatersrand, p. 58, viewed from https://hdl.handle.net/10539/37801

The participants were asked to indicate the functional test used to determine return to function and sport whether they selected the option ‘determine by functional test’. Various assessments such as hop tests, single leg raise, ROM, stability, strength, agility, star excursion balance and vestibular balance tests were included in the survey. In addition, some participants mentioned that the decision is dependent on the patient’s functional level or sport participation. The participants also mentioned under the category ‘other’ that the decision to return to function and sport is dependent on the patient’s pre-operative and post-operative functional level, sport participation or the specific type of sport involved.

The participants had the option to select multiple treatment modalities to manage patients following ACL surgery. Table 4 shows that most of the participants used myofascial release as a treatment modality.

**TABLE 4:** Physiotherapy modalities used to manage patients following anterior cruciate ligament surgery (*N* = 56).

Modality	*n*	%
Myofascial release	41	73
Peripheral manual therapy	35	63
Massage	32	57
Cryotherapy	19	34
Neuromuscular stimulation	18	32
Ultrasound	14	25
Dry needling	13	23
CPM	8	14
Laser	5	9
Blood flow restriction	4	7
Other	13	23

**Total**	**202**	**-**

*Source:* Adapted from Robbertse, C., 2023, ‘Physiotherapy management of post-operative ACL rehabilitation: A cross-sectional survey in Gauteng, South Africa’, Master’s thesis, University of the Witwatersrand, p. 6, viewed from https://hdl.handle.net/10539/37801

ACL, anterior cruciate ligament; CPM, continuous passive movement.

Passive joint mobilisations, muscle energy techniques, patella-femoral and knee joint mobilisation, exercise therapy, proprioceptive neuromuscular facilitation (PNF) and interferential therapy were stated under ‘other’.

Table 5 summarises the period when various exercises in the 0–6-week timeframe commence.

**TABLE 5:** Overview: Exercise prescription in postoperative anterior cruciate ligament rehabilitation.

Timeframe (weeks)	Exercise prescription															
	Isometric		Concentric		Eccentric		Terminal locking		Full extension		CKC		OKC		Cross training	
	*n*	%	*n*	%	*n*	%	*n*	%	*n*	%	*n*	%	*n*	%	*n*	%
0–1	49	88	17	30	10	18	18	32	26	46	7	13	14	25	2	4
1–2	5	9	19	34	8	14	9	16	9	16	16	29	6	11	1	2
2–3	1	2	12	21	10	18	5	9	9	16	12	21	8	14	7	13
3–4	0	0	5	9	14	25	6	11	4	7	9	16	6	11	5	9
4–5	1	2	2	4	4	7	11	20	4	7	5	9	7	13	9	16
6+	0	0	1	2	10	18	7	13	4	7	7	13	15	27	32	57

**Total**	**56**	**100**	**56**	**100**	**56**	**100**	**56**	**100**	**56**	**100**	**56**	**100**	**56**	**100**	**56**	**100**

*Source*: Adapted from Robbertse, C., 2023, ‘Physiotherapy management of post-operative ACL rehabilitation: A cross-sectional survey in Gauteng, South Africa’, Master’s thesis, University of the Witwatersrand, p. 62, viewed from https://hdl.handle.net/10539/37801

CKC, closed kinetic chain; OKC, open kinetic chain.

The majority (70%, 39) implemented an accelerated programme for ACL rehabilitation, with 66% (37) of the participants commencing rehabilitation post-operatively. WB as tolerated (55%, 31) commenced when pain allowed. ROM braces were commonly used for 4–6 weeks (59%, 33) post-operatively. Patients resumed function within 6 to 12 weeks (39%, 22) and returned to sport between 6 and 9 months (39%, 22) following ACL rehabilitation.

The physiotherapy modalities most frequently used included myofascial release (73%, 41), peripheral joint mobilisation (63%, 35) and massage (57%, 32). Regarding exercise prescription, isometric exercises (88%, 49), terminal locking (32%, 18) and full extension (46%, 26) largely commenced during the 0–1-week period, while concentric exercises (34%, 19) and closed kinetic chain (CKC) exercises (29%, 16) commenced during weeks 1–2; eccentric exercises (29%, 14) during weeks 3–4 and cross training (57%, 32) and open kinetic chain (OKC) exercises (27%, 15) commenced after 6 weeks post-operatively.

## Discussion

The accelerated rehabilitation approach involves immediate WB, early progressive knee flexion and extension ROM without restriction and achieving full knee extension. This study shows that only 20% (8) of the participants followed an accelerated protocol as described in the literature (Andrade et al. 2020; Cristiani et al. 2021; Robbertse 2023; Shelbourne et al. 1992). Only one participant followed the non-accelerated protocol as described in the literature. Like Makhni et al. (2016), our results show that diverse protocols are used for post-operative ACL rehabilitation, which causes confusion for therapists. There are no studies to compare in South Africa or globally to establish how many physiotherapists use accelerated and non-accelerated protocols (Robbertse 2023).

Sixty-six per cent (66%, 37) of the participants started rehabilitation post-operatively, contrary to the evidence. The evidence shows that rehabilitation commenced pre-operatively demonstrates better outcomes when compared to rehabilitation that commenced after surgery (Carter et al. 2020; Filbay & Grindem 2019; Giesche et al. 2020; Robbertse 2023; Wilk & Arrigo 2016). Preparing patients physically and psychologically before surgery is important (Robbertse 2023; Wilk & Arrigo 2016), as this aids in reducing post-operative complications, enhances the likelihood of successful return to function and sport and minimises the risk of re-injury. Filbay and Grindem (2019) also advocated for pre-operative commencement of rehabilitation and the aim being reduction of swelling, restoration of ROM and enhancement of quadriceps strength post-operatively. Giesche et al.’s (2020) systematic review supports the idea that pre-operative rehabilitation enhances neuromuscular function and facilitates a return to sports. Carter et al.’s (2020) systematic review indicated improved function, assessed through single-leg hop distance at 12 weeks. Despite this, the review acknowledged the scarcity of high-quality research supporting the assertion that pre-operative rehabilitation leads to enhanced muscle strength, function and return to sports compared to those without pre-operative rehabilitation.

Another fundamental aspect of post-operative ACL rehabilitation is weight-bearing. Mobilisation with FWB commenced as soon as pain allowed by 55% (31) of the participants. Similarly, several authors argue that early WB must be commenced as soon as pain allows (Adams et al. 2012; Andrade et al. 2020; De Carlo and Sell 1997; Jenkins et al. 2022; Naik, Das & Kamat 2019; Robbertse 2023; Wright et al. 2008b). Adams et al. (2012) found that delayed WB and ROM showed poorer subjective and objective outcomes post-operatively, but Fan et al. (2022) advised on delaying early WB and a carefully selected rehabilitation protocol to mitigate the risk of increased ligament laxity and an increase in the tunnel diameter of the graft.

Fifty-nine per cent (59%, 33) of participants reported that their patients use a ROM brace for 4–6 weeks post-operatively, contrary to the evidence (Robbertse 2023). The literature asserts that post-operative bracing is not recommended because of the potential increase in quadriceps muscle atrophy (Andrade et al. 2020; Bordes et al. 2017; Kurtz et al. 2011; Lowe et al. 2017; Naik et al. 2019; Nelson et al. 2021; Robbertse 2023; Wright et al. 2015). Bordes et al. (2017) recommended the use of a brace only in cases where patients exhibit a quadriceps activation deficit preventing the knee from reaching terminal locking or when the surgical graft is deemed fragile (Bordes et al. 2017; Robbertse 2023). Similarly, Naik et al. (2019) reported in a retrospective cohort study that rehabilitation without protective bracing may indirectly guard against re-injury by averting muscle atrophy through early quadriceps muscle strengthening. Andrade et al. (2020) in their systematic review highlighted that scientific evidence does not support the use of post-operative functional bracing and is considered only in cases of more complex injuries involving additional ligaments.

This survey showed that 39% (22) of patients reportedly achieved a return to normal function within 12 weeks post-operatively. In the context of the questionnaire, returning to normal function meant reaching a pre-injury level of function in daily activities, such as resuming school or work and driving. Similar outcomes have been reported in the literature (Andrade et al. 2020; Obermeier et al. 2018), but studies that specifically address return to function in patients undergoing post-operative ACL rehabilitation are limited (Andrade et al. 2020; Obermeier et al. 2018; Robbertse 2023). Obermeier et al. (2018) reported that most patients returned to work by day 11, returned to school by day 7 and resumed driving by day 11. Andrade et al. (2020), however, recommended delaying the initiation of physical work until after 12 weeks.

In this study, ‘return to sport’ was defined as the patient’s ability to participate in their sport and achieve a performance level equal to or surpassing their pre-injury capability. The analysis of results revealed considerable variability in the anticipated time for returning to sport. Regarding return to sport, the finding of this study (Table 3) concurs with the evidence, which also noted a wide range of post-operative return-to-sport timeframes, ranging from 5 to 9 months (Fausett et al. 2022; Robbertse 2023; Sherman et al. 2021). The Multicenter Orthopaedics Outcomes Network (MOON) guidelines stipulate return to sport within 5 to 6 months post-operatively (Wright et al. 2015). Baron et al. (2020), on the other hand, observed that the return to sport commonly occurs 6 to 9 months post-operatively.

The evidence shows that clinical evaluation and functional tests play a significant role in informing clinical decisions regarding the return to sport (Andrade et al. 2020; Fausett et al. 2022; Robbertse 2023; Sherman et al. 2021). Moreover, the literature emphasises the significance of not solely relying on a predefined timeframe but incorporating objective criteria for guidance in making decisions about returning to sports (Andrade et al. 2020; Fausett et al. 2022; Robbertse 2023; Sherman et al. 2021). Andrade et al. (2020) suggested that these criteria should include clinical evaluation using the knee injury and osteoarthritis outcome score (KOOS), the International Knee Documentation Committee subjective knee form (IKDC) or Lysholm scores, functional evaluation using hop test and psychological assessment for readiness to return to sport.

Myofascial release, the most used modality in this study, was found to be more effective than no treatment at all and could serve as a beneficial complement to conventional treatment approaches (Ajimsha & Al-mudahka 2015; Robbertse 2023). Hunt et al. (2010) investigated repeated anterior tibiofemoral glides on knee extension, as a treatment modality for post-operative ACL rehabilitation. Their study assessed the immediate impact of these glides on achieving maximum knee extension during walking and revealed a significant decrease in the extension deficit (*p* < 0.05) with a mean increase of two degrees, corresponding to a 25% improvement in ROM immediately after applying the technique. However, the ROM reverted to its original state after 10 min of rest in seated position. The authors concluded that, despite the widespread use of glide mobilisation techniques in physiotherapy for increasing ROM, there is limited literature evidence supporting their efficacy (Hunt et al. 2010).

Cryotherapy, a modality consistently used by physiotherapists in practice, is effective in reducing post-operative pain (Andrade et al. 2020; Filbay & Grindem 2019; Hart et al. 2014; Kuenze et al. 2017; Robbertse 2023). Thirty-four per cent (34%, 19) of participants in this study used cryotherapy as a treatment modality. The evidence shows that cryotherapy is beneficial for pain reduction (Andrade et al. 2020; Filbay & Grindem 2019; Hart et al. 2014; Kuenze et al. 2017; Robbertse 2023), but it does not help with reducing the effusion around the knee (Filbay & Grindem 2019; Robbertse 2023). Andrade et al. (2020) recommended that cryotherapy only be used for the first 48 h post-operatively.

The evidence shows that exercises in the first 6 weeks of post-operative ACL rehabilitation are to initiate quadriceps strengthening, decrease muscle atrophy and increase neuromuscular activation (Pottkotter et al. 2020; Robbertse 2023; Vidmar et al. 2020). Forty-six per cent (46%, 26) of participants indicated that full knee extension exercises started between 0 and 1 week post-operatively. This is in line with the literature that suggests that it is important to regain knee extension as soon as possible to prevent secondary complications (Ektas et al. 2019; Noll et al. 2015; Robbertse 2023). Isometric exercises most often (88%, 49) started between 0 and 1 week post-operatively. This aligns with the existing literature that states that isometric exercises should be started within the first 6 weeks with the aim of full knee extension by week 4 (Robbertse 2023; Shaw, Williams & Chipchase 2005; Yabroudi & Irrgang 2013). Progressive eccentric quadriceps exercises were also found to be in line with the evidence as most of the participants (25%, 14) indicated that this was started between 3 and 4 weeks post-operatively (Dingenen et al. 2021; Gerber et al. 2009; Robbertse 2023). Open kinetic chain exercises were started after 6 weeks post-operatively (27%, 5) and CKC started 1–2 weeks post-operatively (29%, 6). There is conflicting evidence regarding OKC and CKC exercises, and there is no agreement on when it should be started. More research is required to establish at what point these exercises will be the most beneficial for post-operative ACL rehabilitation (Robbertse 2023). The author believes the observed disparities in the rehabilitation regime may be attributed to individual practices developing unique methods in collaboration with orthopaedic surgeons because of different surgical approaches.

### Limitations

The limitations include a low response rate to the questionnaire, as it was only distributed in one province. Convenience sampling was used, including physiotherapists with various special interests, rather than exclusively those working with post-operative ACL rehabilitation, which limited the establishment of a specific sample size. We did not collect information about the surgical and psychological effects of ACL repair on rehabilitation. For a comprehensive evaluation of the approaches of post-operative ACL rehabilitation, it is crucial to consider not only the rehabilitation component (Robbertse 2023). Other factors to consider include surgical factors, graft placement, healing processes, as well as psychological factors, such as patients’ beliefs and attitudes towards returning to sport.

### Recommendations

High-quality research and randomised controlled trials are needed to determine effective post-operative ACL rehabilitation protocols, physiotherapy treatment modalities and exercise prescription (Robbertse 2023). Further investigation is needed, particularly regarding OCK and CKC exercise prescription, which plays a key role in muscle strengthening. In addition, more research is warranted on ROM progression to prevent complications such as knee stiffness. Other considerations that are important to consider include exploring the impact of surgical factors on the outcome of rehabilitation and the psychological impact of pre-operative education on rehabilitation.

## Conclusion

Despite most participants (70%, 39) implementing an accelerated protocol for post-operative ACL rehabilitation, only a small percentage (20%, 9) strictly adhered to the defined parameters of the accelerated protocol. Variation in rehabilitation approaches was observed. While some rehabilitation strategies aligned with existing literature, such as initiating WB based on pain tolerance and achieving normal function within 12 weeks post-operatively, discrepancies were noted in the timing of rehabilitation initiation and the use of protective bracing. Consensus on the timeframe for patients to return to sport was lacking, and the importance of clinical evaluation, functional assessment and psychological readiness in making such decisions was emphasised. Physiotherapy management incorporated various treatment modalities, such as myofascial release, cryotherapy, neuromuscular electrostimulation, dry needling and peripheral joint mobilisation (Robbertse 2023). Exercise prescription differed greatly, with some agreement with literature recommendations for isometric and eccentric exercises, but no general agreement on when OCK and CKC exercises should be started.
